# Tackling biases in clinical trials to ensure diverse representation and effective outcomes

**DOI:** 10.1038/s41467-024-45718-w

**Published:** 2024-02-15

## Abstract

Professor Sabine Oertelt-Prigione has been working in the field of sex and gender-sensitive research for the last 15 years. Her current work is focused on trying to understand how sex and gender-sensitive medicine can be successfully implemented in research and practice as well as methods to investigate gender in medical research. Dr. Brandon Turner is a resident physician in the Department of Radiation Oncology at Massachusetts General Hospital and Brigham and Women’s Hospital. He has conducted and is involved in numerous studies looking to evaluate race and ethnicity reporting and representation in clinical trials. In this interview for Nature Communications, Sabine Oertelt-Prigione, and Brandon Turner share their knowledge about the biases that can occur in clinical trials and how they can be minimized.

**Figure:**
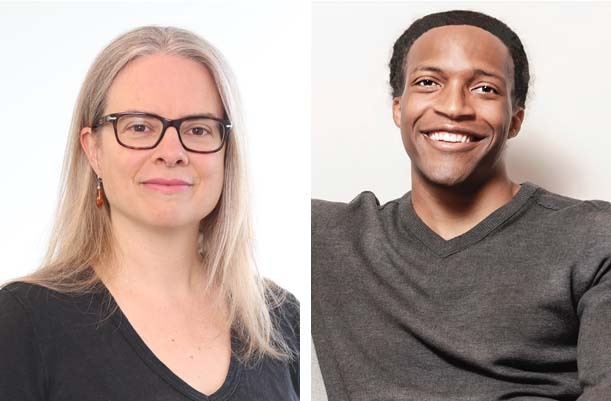
Sabine Oertelt-Prigione (left); Brandon Turner (right)

**1. The reliability of the results of a randomized trial depends on the extent to which potential sources of bias have been avoided. Could you please start with the definition and classification of bias in interventional clinical trials?**

**Sabine Oertelt-Prigione**—If we consider a two-arm trial comparing an intervention with a control group as an example, there are a few examples of bias. S*election bias*, which occurs when individuals with specific characteristics, e.g., comorbidities, are assigned more frequently to one of the two arms of the trial. One could then attribute the trial outcome to the efficacy of the intervention, although the individuals in the control group were simply more ill to begin with. A second bias is *information bias* or *classification bias*, where insufficient or inadequate data is recorded; again, leading to potential misclassification of the trial findings. *Performance bias occurs* when there are systematic differences, for example in how the intervention under study is being performed. If we compare a contraceptive pill that must be swallowed with an intervention that requires some experience, e.g., the use of a female condom, results could be biased not due to differences in effectiveness, but differences in performance of the intervention. Another bias is *detection bias*, which is associated with differences in how the outcomes under study are being determined. In fact, certain expectations by the study participant or the trialist might lead to heightened attention to certain outcomes that are in line with the original expectations impacting the likelihood of detection*. Attrition bias* occurs when participants abandon the two groups at different rates due to unidentified factors. *Confounding bias*, on the other hand, occurs when the trial effects are attributed to an intervention, although they are caused by an unidentified factor associated with exposure and outcome. A last and important form of bias is *reporting bias*, which is linked to how data is presented and reported in publications. In our current system authors are rewarded for novelty and positive findings, leading to a potential underreporting of negative or confirmative results.

**Brandon Turner**—As highlighted by Sabine, there are so many sources of potential bias within a clinical trial. I find it helpful to structure them along two dimensions. The first dimension is whether the bias impacts the internal validity (i.e., does the trial measure what they purport to measure) or external validity (i.e., can the trial results be applied to patients in the real world) of the study. Most of the biases we discuss impact the internal validity of a study, however reporting bias primarily impacts external validity. The second dimension is where the bias emerges within the lifecycle of a trial. At the study design stage, biases that can emerge include selection bias (e.g., poor randomization or skewed inclusion/exclusion criteria), confounding bias (e.g., not measuring or stratifying by potential confounders), or subtle biases due to flawed treatment arms (e.g., using a weak or flawed comparison arm to demonstrate the efficacy of the experimental intervention). At the trial conduct phase, differences between providers or study facilities in how data is collected or recorded (possible sources of classification bias or detection bias) or in how interventions are administered (possible source of performance bias) can produce bias. This also includes differences between treatment arms (e.g., attrition bias). Finally, at the data analysis and reporting stage, biases in analysis (e.g., use of suboptimal endpoints or not controlling for confounders) or reporting bias (e.g., selective reporting of specific results) can emerge. Additionally, while it’s not a form of bias within a specific trial, there can be bias in the wider clinical research enterprise in terms of which diseases and which therapies get funding for a clinical trial at all.

**2. What steps are conventionally taken to avoid such biases? Are there firm regulations or more suggested guidelines on how to avoid bias and are such regulations/guidelines country- and/or context-specific?**

**Sabine Oertelt-Prigione**—Several strategies have been developed to avoid most of these biases and are now common practice when executing pharmacological and intervention trials. One measure is *randomization*, which is the process of randomly assigning participants to one of the—two or more—groups under study to avoid selection bias. A second strategy against selection and detection bias is *blinding*. This means that neither the participants nor the investigators know which trial arm the person has been assigned to. Furthermore, trials should follow an *intention-to-treat design*, meaning that individuals will be assigned to one of the two groups and that their information will be analyzed as part of that group regardless of the outcome. This should maintain the benefits of randomization and reduce selection, detection, and attrition bias. Last, all trials should be officially registered in one of the national or international *repositories* available and ideally, detailed trial protocols should be published. After trial completion, publication of positive as well as negative results should be ensured to avoid publication bias.

Regarding regulations, there are both national and international guidelines on how to conduct trials that detail which requirements need to be fulfilled. While many requirements are binding, the inclusion of women and men as well as racial or ethnic minorities in clinical trials is not standardized. In 2016 the US Federal Drug Agency (FDA) published the guidance titled *Collection of Race and Ethnicity Data in Clinical Trials*, which encourages sponsors to “*enrol participants who reflect the demographics of clinically relevant populations with regard to age, gender, sex, race, and ethnicity*”. The European Medicines Agency (EMA) uses a similar wording in its EU Clinical Trial Regulation No 536/2014 stating that *“unless otherwise justified in the protocol, the subjects participating in a clinical trial should represent the population groups, for example, gender and age groups, that are likely to use the medicinal product investigated in the clinical trial*” and “*non-inclusion has to be justified*.*”* Several recommendations can also be found under the umbrella of the International Council for Harmonization of Technical Requirements for Pharmaceuticals for Human Use (ICH), which brings together international regulatory authorities and the pharmaceutical industry. However, while these regulatory bodies offer recommendations about the consideration of women and minorities in clinical trials, they define no binding benchmarks.

**Brandon Turner**—There are simple and standard practices that can be used to avoid some of these biases (e.g., randomization, blinding, pre-registration of analytic protocols) which are outlined above. However, steps to address the more nuanced biases are challenging and often come with their own trade-offs which can increase the difficulty or cost of running the trial. The increasing complexity of trials, including now even Phase 1 trials, has resulted in significant cost pressure for each touchpoint with each participant a trialist hopes to accrue. The number one cause of trial failure is inadequate accrual, so we must take seriously that the pursuit of idealized designs doesn’t preclude valid studies with sensible designs. For example, a growing trend in Oncology is to utilize “Physician’s Choice” as the comparison arm (i.e., physicians choose what treatment they think is best) to the experimental drug. While this freedom reduces the likelihood of physicians feeling forced to use what they consider a suboptimal control group, and thus likely improves their willingness to participate in the trial, it introduces a new possible confounding factor into the analysis.

There are not many regulations or governmental guidelines that impact most of these biases given the scientific nuances between trials. Consequently, most of the government’s involvement in clinical research is instead in the realm of patient safety (e.g., requirements for informed consent, institutional review boards, results reporting, adverse event reporting, and good manufacturing practice). Beyond safety, a lot of a government’s ability to influence trial biases is through their authority to approve drugs and devices. For example, the Food and Drug Administration (FDA) publishes guidance on acceptable biomarkers and surrogate endpoints for various clinical entities, which trialists are of course strongly incentivized to follow if they desire FDA approval. An area of long concern but which has more recently gained traction is the need to improve the inclusion of women and minorities in clinical trials.

**3. We know it is important to have accurate and diverse representation in clinical trials. How are study participants with different sex and gender represented in current clinical trials? How are non-binary study participants represented?**

**Sabine Oertelt-Prigione**—First, it is important to define the terminology. Sex captures biological characteristics, such as genes, hormones, and anatomy, which can distinguish individuals into the categories of female, male, and intersex. Gender is a distinct multidimensional concept that includes identity, societal norms, and the power dynamics between individuals interacting with each other. Clinical trials rarely collect data about gender, and if they do, the variable recorded is usually gender identity, i.e., being a man, a woman, non-binary, queer, gender fluid, and many more. Gender norms and relations are usually not investigated. Most trials focus solely on sex, although a two-step approach registering sex at birth and current gender identity could easily expand this information to include gender. The majority of participants in currently performed trials and surveys identify as men or women, and if the trial population is relatively small, statistical challenges might emerge when a limited number of participants identify as another gender. To guarantee statistical power and allow the detection of meaningful associations, a selective oversampling of certain gender identities could be considered upon trial recruitment, although this is still rarely done. Researchers should be aware that enquiring about sex and gender during a trial will be relevant to answering different questions or even different angles of the same questions. For example, if I want to investigate differences in side effects, I might have to know about both sex and gender. In fact, biological differences in enzymatic metabolism might be associated with sex differences, however, differences in reporting of side effects and being listened to might have a strong gender component.

**4. How are study participants of different races and ethnicities represented in current clinical trials?**

**Brandon Turner**—There are multiple lenses through which we can look at representation. Often when we talk about a patient’s race and ethnicity, we’re blending two concepts. The first is their genetic heritage, which may tell you something about the way patients from a particular group may react differently to a disease or intervention as a result of commonly inherited genes. The second is their sociocultural context, which brings in all the behavioral, cultural, and environmental factors that we also know impact people’s health. The latter is more directly aligned with the social construct of race and ethnicity. We very poorly understand representation by genetic heritage right now, mostly because of the cost to acquire this information, but no doubt there also would be ethical and privacy hurdles.

Globally, there has been a major shift with the emergence of clinical research emerging from East Asia, particularly China. This boosts Asian representation in trials. For example, the recent PD-1 inhibitor Sintilimab (Eli Lily) was developed using enrolment entirely from Chinese facilities. In contrast, its major predecessor, Nivolumb (Bristol-Myers Squibb Company) was developed using enrolment across 14 different countries in North America, South America, Europe, and Asia. African involvement in clinical trials has been minimal comparatively, which limits a potential source of data from patients with African ancestry.

However, most of the research on demographic representation has emerged from the US, which also runs the most clinical trials currently. There, recent data suggests that all minority groups are underrepresented relative to their population. When comparing to the US population, the data suggests the largest disparities may in fact be within Asian American and Latino-American communities. However, when disease burden and epidemiology are taken into consideration, the largest disparities are seen in Black Americans. This relationship can be further nuanced when you consider geographic distribution and socioeconomic status.

**5. What are the consequences of sex and gender bias in clinical trials?**

**Sabine Oertelt-Prigione**—In my opinion, sex- and gender-sensitive medicine is about three core principles: equity, safety, and change. *Equity* in terms of offering the best possible access and care to all individuals regardless of their sex, gender, or any other individual characteristics. *Safety* in the form of respect and acceptance as well as the availability of robust information about the effectiveness of a therapy and the absence of side effects. And *change*, because the achievement of the former two core principles will require systemic changes in the way we currently practice medicine.

Sex and gender bias substantially threatens the safety of therapy, as a biased study will limit the availability of reliable information about effectiveness and potential side effects for all participants. This can be due to inadequate recruitment, lack of statistical power at the subgroup level, or reporting bias. It is well documented that female patients report more side effects for most drugs, ranging from cardiovascular medications to chemotherapy. We still have very little information about the impact of side effects in gender minorities, although this might be an important area of study for example for individuals taking gender-affirming hormones that might interact with other medications. Furthermore, safety is not solely a question of pharmacodynamics but also of health equity. Barriers to access to care, such as economic constraints and various forms of discrimination, can prevent groups of individuals from obtaining the care they need and deserve.

As mentioned before, clinical trials mostly focus on sex, yet ignoring gender can be a significant source of bias, especially upon recruitment. People might not feel appropriately addressed by our currently developed gender-neutral recruitment and information materials or might not feel sufficiently reassured when invited to participate. Furthermore, being a gender minority can be a source of exclusion from trials for several reasons. For example, individuals might not be willing to engage with the healthcare system due to prior experiences with discrimination and lack of safety, or trialists might be actively excluding them due to statistical concerns.

The Snapshot project by the FDA (https://www.fda.gov/drugs/drug-approvals-and-databases/drug-trials-snapshots) offers a user-friendly overview of, among other things, of sex differences in efficacy and side effects. Most listed pharmaceuticals appear to demonstrate no sex differences in the incidence of side effects. Nevertheless, as the agency itself cautions, robust conclusions cannot always be made. This can be due to differences in the recruitment of female and male participants or to the insufficient size of the sex-specific subgroups for robust statistical testing if the expected differences are small.

**6. What are the consequences of race and ethnic bias in clinical trials?**

**Brandon Turner**—I believe there is an equity dimension and a scientific dimension here. From an equity perspective, clinical trials have some intrinsic value in themselves. They offer access to the latest therapies and often patients receive extra diligent care, which is partially why trial outcomes can sometimes be rosier than seen in the “real world”. A fair clinical research apparatus should endeavor to provide access to this limited resource as evenly as possible except where the science dictates otherwise. Thus, bias away from equal representation in trials denies equitable access to the intrinsic benefits of clinical trial participation. This consequence is intuitive to most observers.

From a scientific perspective, poor race and ethnic representation means there may be insufficient power to detect salient differences between demographic groups in terms of an intervention’s efficacy or safety. This risk is especially important in the precision medicine era where we are increasingly reliant on exploiting small molecular differences to achieve therapeutic or diagnostic benefits. Interestingly, this risk is often less intuitive to clinicians and scientists precisely because there has historically not been enough research with minority populations which could consequently enable one to observe these subtle differences in the first place. Today there is a growing body of evidence that demonstrate the direct impact of poor representation of race and ethnic minorities on findings ranging from the efficacy of biomarkers for the efficacy of immune checkpoint inhibitors to the likelihood of off-target effects with CRISPR gene editing. In the past 15 years, 10–20% of new molecular entities approved by the FDA have demonstrated differences in drug disposition and pharmacokinetics across different sub-populations. Our ability to safely detect and manage these disparate effects is dependent on having adequate representation with which to study them. However, scientifically speaking, it is not clear what level of representation is adequate.

**7. What are community-based clinical trials and how do they differ from standard randomized controlled clinical trials?**

**Sabine Oertelt-Prigione & Brandon Turner**—Instead of constructing a trial in a clinical setting that only partially resembles the lived reality of the participants, community-based trials aim to move the same methodological rigor offered in a hospital setting into the community. For example, the same pharmacological intervention usually administered in the hospital is now moved to a doctor’s practice or a healthcare center in the community. Overall, community-based trials are designed to build on the trust and long-term relationships that exist between community-based providers, such as nurses, physicians, and other health professionals, and use these bonds to improve access and recruit a trial population that resembles the final target group as closely as possible.

The community-based clinical trial approach overlaps with a rapidly growing movement to pursue decentralized clinical trials (DCTs). Here the focus is similarly on enabling trial participation outside of the traditional hospital centers. DCTs can and typically do leverage community-based clinics. However, they can also be designed to bypass the need for any in-person contact at all. Instead, patients are managed using a combination of virtual visits and various interventions delivered digitally or through the mail. Hybrid models are also possible where some care is still obtained at the main site, but most care is still obtained in the community.

The other interpretation of community-based trials is making the entire community the unit of observation. Instead of focusing on a single individual, the entire household, neighborhood, or village is used as a unit of analysis. This approach is employed in a growing number of public health and epidemiological trials, especially in low- and medium-income settings. It can work well to study interventions that address groups rather than individuals, such as fluoridation of drinking water, family planning counseling, or community-based interventions for cardiovascular disease prevention. This type of intervention is however not suited to study disease-specific pharmacological interventions, since these are by definition targeted at single individuals.

The transition to community-based trials was accelerated by Covid-19. In the early days of the pandemic, many patients were unable or unwilling to go to main sites for in-person visits. The number of new Covid-unrelated trials initiated during the first months of the pandemic fell by half while a large number of trials had to delay completion due to disruption of normal trial activities. As seen in many other sectors of the economy, the incentive to find creative ways to resume trial activity increased interest in using virtual and local solutions. The FDA has assisted this transition with multiple guidelines to promote not just DCTs but also the development and distribution of digital health technologies which are crucial to these new community-based models.

**8. How can community-based clinical trials work to reduce bias and improve diversity, equity and inclusion in clinical research?**

**Sabine Oertelt-Prigione**—Reaching people in their communities can help in reducing some of the hurdles related to hospital-based trials. Transport times and costs, availability of care for children, and loss of economic means can represent barriers to attendance at the hospital. Physical barriers during the journey to the facility can limit access for individuals with disabilities. Also, mistrust due to negative prior experiences with the healthcare system, such as discrimination, disrespect, or inability to make informed choices, can prevent people from participating. Furthermore, reaching participants in an unsecured housing situation or with a history of substance use is complex if a distant location has to be reached.

Community-based care offers the opportunity to build trust over periods of time, increasing the opportunities to engage a more diverse, representative, and potentially difficult-to-reach population. A community setting can improve recruitment by making use of multiple community locations to promote a trial, such as supermarkets, barbershops, or drugstores. Information can be offered in different languages and could be developed in a co-creative process with the community to guarantee tailoring and access to the target group. Another very important point might be the representation through the healthcare providers themselves. If the trialist is a person of the same racial, cultural, or religious background as the participants, trust might be easier to establish, especially if minority groups are being recruited.

**Brandon Turner**—One of the major benefits of community-based clinical trials is the ability to recruit patients beyond those who are conveniently located near a large hospital. In theory, this would permit communities with underrepresented populations to be more easily accessed, thus improving diversity and reducing bias from non-representative trial cohorts. In practice, many academic centers are already located in diverse, high-density metropolitan areas yet still often enroll non-representative cohorts. Similarly, industry-funded trials that rely on a network of healthcare centers often recruit patients from the same set of centers from which they’ve had good enrolment in the past. These are less common in underserved communities. For community-based clinical trials to fully realize the promise of improving diversity, there will need to be deliberate efforts to ensure they are actually recruiting from communities with underrepresented populations, and that those populations are actually making it into the trials.

Here trialists can likely learn from public health practitioners, who have much more experience in trying to interface with communities (e.g., collaborating with community leaders, faith leaders, clinical leaders, and major employers in order to help improve messaging and outreach) to improve the uptake and success of health-related interventions. Few trial sponsors will have the expertise, and many will not have the resources, to manage such relationships. The best strategy is still unclear and actively being pursued by both private sector entities, like clinical research organizations (CROs), and academic and advocacy organizations.

**9. Are there specific challenges in designing and reporting community-based trials? For example, with respect to participant recruitment or ethical oversight?**

**Sabine Oertelt-Prigione**—In any trial, we try to minimize unaccounted influences in order to robustly extrapolate the effect of the intervention. In practice, this means trying to control for different aspects, e.g., individual or disease-related factors, as well as environmental influences. Individual factors can be sociodemographic characteristics, such as age, sex, and education. Disease-related factors can be chronic therapy or the presence of co-morbidities. Environmental factors can be related to housing or safety within a community. A hospital setting allows to minimize some of the environmental influences during the trial, and allows for better control of individuals and disease-related ones. Recruitment in the community can potentially reduce selection bias and increase the representativeness of the enrolled population. But it may also pose logistical challenges.

In community-based cluster trials, households or entire communities are treated as units of investigation, rather than a single participant. In this case, the randomization process can be challenging due to unplanned sharing of information between the intervention and control group, or because randomization units might not accept the randomization process. Furthermore, the researched units will be limited in number and thus pose statistical challenges, as the sample size may be too small. When performing a community-based pharmacological trial, procedural accountability has to be maintained and data safety guaranteed, both of which be challenging if multiple providers are involved.

**Brandon Turner**—Community-based trials bring a new set of challenges. Before even considering participant recruitment, many community practices simply lack the clinician experience or the equipment and resources necessary to participate in increasingly complex modern trials. Training personnel (e.g., completing regulatory paperwork, discussing trial opportunities with patients, properly delivering investigational drugs, and obtaining biospecimens) and acquiring the necessary equipment (e.g., refrigeration and storage for biospecimens and investigational products, specialized devices required for measurements or treatment delivery, etc) require additional startup investment from trial sponsors, though many of these costs become significantly reduced once the site is established. Coordinating centers that assist with training and integrating information from multi-site trials have grown within both public and private institutions. However, data governance at community sites is an additional challenge as data must be either securely stored (which requires additional equipment) or transferred to a main site, all while ensuring patient privacy and preventing unauthorized access. Digital platforms are emerging to address some of these concerns, though the market is still quite nascent.

The primary concern for most sponsors is ensuring the integrity of findings. Meaningful effect sizes can be obscured if measurement precision deteriorates. This may be a challenge for designs that rely on patient self-assessments (whether subjectively or by operating supplied equipment like wearable biometrics) or remote clinician evaluations. Even evaluations by in-person clinicians in the community who are less experienced could potentially exhibit greater inter-rater variability than would be seen in a centralized design at a large health center. These challenges extend also to the monitoring of potential adverse effects and have drawn oversight concern and attention that the focus on providing convenience and access does not come at the expense of vigilance and patient safety.

**10. Can you elaborate on recent success stories that we should consider as stepping stones to inclusive trials?**

**Sabine Oertelt-Prigione**—When looking at examples about gender, some government agencies have stepped up and developed excellent materials. For example, the division of AIDS (Acquired Immunodeficiency Syndrome) at the National Institutes of Health (NIH) has developed very helpful resources for inclusive gender-sensitive trial design, ranging from communication to analysis. These are good examples when working with a very diverse participant population. In the field of gender-sensitive prevention, the British example of the Football Fans in Training (FFT) trial is also an excellent example of the consideration of masculinities in developing lifestyle interventions. The authors spent many years investigating the underlying concepts of masculinity that led to unhealthy behaviors and identified gender-specific barriers to participation. Based on these findings, they designed a very successful community-based trial, which set an example for other initiatives in Europe. In the cardiovascular field, we have also seen important progress in the last decade in terms of the inclusion of female participants in clinical trials. Recent analyses have highlighted that the inclusion rates for most conditions are representative of the prevalence in the general population.

Reporting of sex-specific side effects is, however, still not the norm. For example, in our recently published paper about the consideration of sex and gender in registered COVID-19 trials, we have found that only about 20% of the published trials included some form of sex-specific analysis (Brady et al. Nat Comm 2021—doi: 10.1038/s41467-021-24265-8). The studies that included such analyses, used different approaches, ranging from including sex as a variable in forest plots of efficacy, sex-disaggregated reporting of results, and disaggregated reporting of side effects. Very large trials were mostly setting the example by providing adequate subgroup analysis.

**Brandon Turner**—The recent Pfizer and Moderna Covid-19 studies achieved minority representation that exceeded that seen in typical Phase 3 and especially vaccine trials. Crucially, these trials relied on networks of community sites (across multiple nations) which undoubtedly played a role in their diverse cohort. While the pandemic certainly created a unique and well-publicized recruitment environment, the logistics and success in leveraging community sites are very promising.

The National Cancer Institute (NCI) has tried to encourage community engagement with clinical trials through a number of initiatives. The NCI Community Oncology Research Program (NCORP) is a national network of community sites. Crucially, 30% of their community sites are designated as Minority/Underserved Community Sites, which are sites with a patient population comprising at least 30% racial/ethnic minorities or rural residents. Many of the NCI network trials involving genomic sequencing and precision medicine approaches draw a portion of their patients from NCORP sites. Recent data show that these trials have better representation than similar pharmaceutical company-sponsored trials. An important element of NCORP is demonstrating that it need not be “all or nothing”—trialists can draw on a variety of community and academic sites as a way to achieve representative samples. However, care must be taken that data generated in such disparate practice settings is equivalent in quality and can be combined without introducing bias.

**11. What can doctors, regulators, funders, and patients do to increase diversity in clinical trials? Do you envision a role for publishers as well?**

**Sabine Oertelt-Prigione**—Regulators have a fundamental role in defining rules for inclusive trial design. Regardless of the funding source of a study—public or private—any medicinal product will have to pass regulatory testing. Hence, regulators´ rules apply to everyone seeking to obtain market approval for a pharmaceutical product or device. Funding agencies have significant power over the execution of inclusive trials in a landscape where external funding is necessary to conduct a trial. They have the ability to combine the availability of funding with grounding principles and rules, encouraging inclusive trial design. Patients have also become more vocal stakeholders over the years, and have been essential in diversifying the conversation about clinical trials and inclusive healthcare. Their voices are important, both as organizations and as individuals in the doctor´s office.

Publishers could also play a fundamental role in setting basic requirements for the manuscripts that they are making available to the public. In my conversations with regulators, I am often told how pharmacological dossiers contain information about sex differences in efficacy and side effects, yet I rarely find this information in the published literature. This discrepancy could be avoided if mandatory recommendations were applied by the publishers and enforced upon publication. Whenever I submit a manuscript, I am asked to comply with numerous layout requests, yet no formal rules apply regarding analytical inclusiveness. Open data sharing is an important step in the right direction, but I could still publish a manuscript today without reporting any sex-disaggregated analysis or any sex-disaggregated side effects of a drug or intervention. The International Committee of Medical Journal Editors (ICMJE) has developed specific recommendations, and the Sex and Gender Equity in Research (SAGER) are available to fill this gap.

**Brandon Turner**—This is admittedly a massive topic but I’ll try to touch on highlights. Concerns about minority mistrust are well-publicized and also well-founded given the history of medical and scientific misconduct, particularly toward Black Americans. However, research has repeatedly shown that minorities are just as likely as White individuals to participate in a clinical trial when asked (and in some cases are more likely). So, I think patients are willing and ready to do their part, and physicians must ask, and ideally invest in learning best practices for discussing the benefits of trial participation. Academic physicians and hospitals could play a unique role in expanding the network of physicians who recruit patients into trials—especially for physicians who see underrepresented populations in community settings.

Regulators have a few levers that can be pulled. The FDA has issued guidelines in the past and last year enhanced this effort with new draft guidance for the industry which recommends that sponsors draft a plan for how the trial will enrol a representative population into their clinical trial. The FDA then recommends that this be submitted in the application for an investigational new drug (IND) or for investigational device exemptions (IDE). Notably, it’s not a requirement, there’s no penalty, and the FDA leaves open what are reasonable mechanisms to both define and achieve this goal, though some structured suggestions are offered. Stronger FDA guidance or requirements here would likely compel greater effort.

The FDA also could consider creative, positive incentives like fast-tracking evaluation of IND and IDE where studies featured representative cohorts. Other agencies like the Centers for Medicare & Medicaid Services (CMS) can similarly fast-track decisions on which investigational products will be covered to prioritize those with supportive data from representative populations. Crucially, many of the barriers to participation in a trial could be ameliorated with financial compensation to trial participants for transportation, lodging, and childcare. Agencies could help defray this cost for trials that meet certain diversity criteria. In addition to supporting patients, CMS could expand reimbursement codes to cover more clinical trial activities, particularly including research support staff which are increasingly the dominant variable cost associated with trials. This would benefit academic medical centers and also likely accelerate participation of community centers which typically lack the infrastructure and personnel for frequent participation in trials.

Funding agencies can also increase support for cost-related burdens as discussed above. However, there is a dearth of knowledge on best practices that can ensure optimal allocation of limited funding resources. There needs to be funding for analytical and implementation studies that evaluate the effectiveness of various strategies for measuring and achieving representative clinical studies. This is crucial, as there remain many uncertainties shared across stakeholders (including regulatory, industry, and academic) that risk creating arbitrary and potentially wasteful goals (e.g., how much representation is “enough”, which epidemiological or published data is the appropriate standard for evaluating disease burden, which factors are causal drivers of non-representative trials and what are their relative magnitudes of effect relative to other modifiable factors, etc).

There is a strong history of publishers’ role in improving clinical trial transparency and quality. In 2005 when the International Committee of Medical Journal Editors (ICMJE) required clinical trials published in member journals to have registered their trial in a clinical trial registry (such as ClinicalTrials.gov) before patient enrolment, it helped to topple common practices that frequently did not disclose details about trials’ design or existence to the public. Publishers should require and help to standardize the reporting of clinical trial patient demographics (which is often non-standard or missing entirely from publications) including cross-tabulation for the intersection of race, ethnicity, and sex. Analyzing results stratified by race is not feasible for many initial studies given the available power. However, collecting and publishing this information (e.g., in supplements) provides the opportunity for pooled secondary analyses. The effort to enroll underrepresented participants is squandered if their data is not accessible. Publishers also should encourage and promote high-quality and innovative studies that aid our understanding and improvement of trial enrolment dynamics.

